# Direct and Indirect Management Models in Public Health in the Framework of Mental Health

**DOI:** 10.3390/ijerph20032279

**Published:** 2023-01-27

**Authors:** Elena Puerto-Casasasnovas, Jorge Galiana-Richart, María Paola Mastrantonio-Ramos, Francisco López-Muñoz, Alfredo Rocafort-Nicolau

**Affiliations:** 1 Departamento de Empresa, Facultad de Economía y Empresa, Universitat de Barcelona, 08034 Barcelona, Spain; 2 Departamento de Contabilidad y Finanzas, EAE Business School, 08015 Barcelona, Spain; 3 Facultad de Ciencias de la Salud, Universidad Camilo José Cela, 28692 Madrid, Spain; 4 Departamento de Contabilidad y Finanzas, La Salle, Universitat Ramon Llull, 08022 Barcelona, Spain; 5 Departamento de Economía, EUNCET Business School, 08016 Barcelona, Spain; 6 Unidad de Neuropsicofarmacología, Instituto de Investigación Hospital 12 de Octubre (i + 12), 28041 Madrid, Spain; 7 Portucalense Institute of Neuropsychology and Cognitive and Behavioural Neurosciences (INPP), Universidade Portucalense, 4200-072 Porto, Portugal; 8 Departamento de Economía Financiera y Contabilidad, Universitat de Barcelona, 08034 Barcelona, Spain

**Keywords:** mental health, gross domestic product, per capita expenditure, public ownership, agreement

## Abstract

This article analyzes the relationship between per capita expenditure and financial and macroeconomic variables in the framework of mental health, in regions where the prevailing system is public healthcare governed by the state and in regions where the prevailing system is that of public ownership. The period 2006–2017 was analyzed. A simple linear regression analysis was carried out to determine the relationship between the expenditure per inhabitant and a series of relevant variables such as asset turnover, cash flow, and expenditure as a percentage of gross domestic product (GDP), applying statistical tests to validate the study. In regions where public–private co-financing prevails in the health system, two crucial variables to measure per capita expenditure on mental health were GDP per capita and cash flow of mental health providers. In the regions where management is direct, the crucial variables were asset turnover of mental health providers and expenditure on mental health as a percentage of GDP per capita. These elements are key to determining how to develop public investment policies in hospital systems in the different regions of Europe and the world.

## 1. Introduction

Some of the most representative items of public expenditure worldwide are those derived from health and education [1]. Focusing on the health field, problems derived from mental health are clearly on the rise in our society [2,3,4,5]. Mental disorders have a very high impact not only in economic terms, but also affect other areas of life such as family, social relationships, and work [6]. This situation is aggravated by the social exclusion of people who suffer from these pathologies [7].

Unlike other pathologies, such as cardiovascular and oncological diseases, mental disorders tend to have a chronic course and manifest at an early age [8]. Seventy percent of mental illnesses diagnosed before the age of 18 tend to persist for decades [9].

This situation worsens if the sick population does not receive basic treatment. As reported by the World Health Organization (WHO), approximately 75% of the population suffering from mental disorders is not adequately treated [10]. Furthermore, according to the WHO, the economic and social repercussions derived from the prevalence of mental disorders are of considerable magnitude. Not only should the economic cost derived from the health system be considered, but also other intangible costs whose quantification is more difficult to calculate; for example, the reduction in the quality of life and the emotional stress suffered by both people who have a mental illness and their families [11].

In this sense, a WHO report indicates that the economic impact of mental illness is reflected in personal income and in the ability of individuals or their families to work and make productive contributions to the national economy. This report also establishes that the use of care services and the support received by people with mental illnesses also increases [12].

After a study published in *The Lancet Psychiatry*, the WHO argued that the investment made in the two most prevalent mental disorders, depression and anxiety, had a return of 400%. Therefore, there are sufficient arguments to increase investment in mental health services in all countries, regardless of income level. More specifically, this report stated that “Every US$1 invested in scaling up treatment for depression and anxiety leads to a return of US$4 in better health and ability to work” [13].

As mentioned, the incidence of mental disorders is increasing considerably [14]. Between 1990 and 2013, the number of people who suffered from depression or anxiety increased by approximately 50%, with mental disorders representing a worldwide non-fatal disease burden of 30% [13]. Despite this, according to the survey carried out by the WHO Mental Health Atlas, the investments that the authorities make for the treatment of mental illnesses are much lower than what is needed [15]. States spend, on average, 3% of their budget on mental health. This number ranges from 1% in lower-income countries to 5% in higher-income countries [15].

According to Almeida-Filho [16], “economic and social inequalities end up producing new forms of health inequities”. Along the same lines, considering the economic impact of poor mental healthcare, a study carried out by the World Economic Forum and the Harvard School of Public Health [17] estimated that “the global impact of mental disorders, in terms of loss of productivity, will amount to 16.3 billion dollars between 2011 and 2030”.

Mental disorders often lead to a difficult economic situation. The loss of productivity for the patient and their family environment represents a loss for society since disability benefits are sometimes received, partially offsetting the loss of income. Therefore, the loss is shared between the individual and society [18]. Therefore, it is suggested that the investment in health by the state should be greater [19].

The amount a country spends on health and the rate at which it can grow over time are influenced by social and economic determinants [20]. It is also an indispensable factor for society and the financing and organizational structure of the health system itself. In particular, there is a strong relationship between the general level of income of a country and how much the population of that country spends on healthcare [14]. Thus, health systems are struggling with rising health expenditures [21,22,23].

The COVID-19 pandemic has revealed a certain fragility of the health and social systems, leading to greater inequality in the living conditions of a large population [24]. Mental disorders represent a cost of 2.2% of GDP [25,26].

An indicator that reflects public expenditure is health expenditure per inhabitant (per capita) and expenditure as a percentage of GDP in each region [27,28,29]. However, health systems are managed in different ways, as their classification has always been articulated by their financing mechanisms [30], or on the basis of the existing contractual relationships between them and healthcare service providers.

A decentralized model is that in which regional health management systems are included; that is, those management systems in which regulation, co-financing, and operation are delegated to regional authorities [31]. The main characteristic of this decentralized healthcare system is that the regional authorities assume a very important role [32]. Virtually universal coverage is provided at the point of service [33] and the healthcare provided is primarily financed with public funds from national and regional taxes [34,35,36,37,38].

The health management model can be public, private, or mixed, as long as it guarantees equal conditions for all citizens. Thus, public administrations own the management of the public service, and this can be managed directly through their own organization and available means, or indirectly.

Regarding indirect management, the technique most used in health to contract services is the agreement, which allows contracting public or private companies that will provide the public health service.

In public healthcare, offers must be adjusted according to demand, as the market has very particular characteristics and is regulated in a certain way. Thus, it is a matter of defining the structures of the public offer. In this work, we will try to determine what types of centers are needed and where they are needed to be able to satisfy the needs of the users of said system.

Following this line, the agreement formula allows the administrations that own the service to equip themselves with fixed assets and personal and technical assets of a company, generally for profit, already established through the consideration of financial compensation for the provision of said service. When the administrations that own the service manage it directly, we see non-profit companies that, therefore, are publicly owned.

Therefore, in those regions where agreements are used, the supplier companies seek to make a profit. On the other hand, in those regions where the management is direct, no profit is sought.

From a financial point of view, investment is understood to be the assets that a company has [39] (in the present case, the public mental health system). In order to consume the assets, sources of financing are needed, these being liabilities and net worth [40]. Liabilities represent the obligations arising as a result of past events. The net worth includes the contributions made by the partners or shareholders, as well as the profits generated and not distributed.

Liabilities and equity represent the financial structure since these contain the sources of financing that the company uses to finance its investments. One of these sources available to the company is self-financing [41], which corresponds to funds generated by the company and reinvested in itself to stay in operation and/or expand [42]. In other words, it is the part of the cash flow that returns to the company as a source of financing. The greater the company’s ability to generate resources that are reinvested in itself, the greater its financial independence from third parties [41]. Cash flow is the net profit plus the amortizations, understanding these as the consumption that is made of the asset elements, either by the use that is given to them, by the passage of time and/or due to technological obsolescence of all those assets that have a useful life of more than one year.

In order to evaluate the evolution of a company, a turnover ratio is used that relates elements of financial information reflected in the financial statements [43]. The purpose of this ratio is to analyze asset performance. It is calculated by relating the asset to sales and it is of interest that the resulting value is the highest possible. In the case at hand, we understand sales as the billing/income that occurs in the public health system.

To summarize, in Table 1 we provide a description of the variables used in our analysis.

The objective of this work was to determine the influence that certain macroeconomic and financial variables have on the expenditure per capita with regard to mental health in public healthcare, depending on whether the health system in the region is publicly or privately owned, that is to say, managed by agreement.

This study is relevant given the lack of similar updated works. It will also help us detect possible intervention areas to achieve efficiency and equity for health services, whether under public title or via concerted effort within a mental health framework. Based on our literature review, we formulated the following hypotheses according to the general study objectives.

**Hypothesis1 (H1)**. *Differences in function are observed between a health system under public title and a co-managed system.*

**Hypothesis2 (H2)**. *Economic activity influences both models equally*.

**Hypothesis3 (H3)**. *Business management is a determining factor for both models*.

## 2. Methodology

For the present work, two communities in Spain were considered. The data were obtained from the CatSalud database and the Central de Balances, which was created in 1991 with the aim of being an instrument of consensus between the hospitals and the public centers of the hospital network in Catalonia and the Catsalut [44]. Economic-financial information was obtained from the health provider centers. In addition, the information was disaggregated by a mental health center.

The most relevant variables in financial terms were included, such as income and the various results presented by summaries from mental health centers, as well as the most relevant figures for the investment needed and funding sources [43].

For the demographic and macroeconomic variables, we considered available data with homogeneous criteria to use in the present study [45,46].

The data collected from regions where publicly owned centers prevail were obtained from the general budgets of the Basque Government [47]. The annual accounts of the Mental Health Network, which manages the health service, were also collected. Likewise, the macroeconomic and socio-health data were extracted from different sources, such as the National Statistics Institute [48] and the Catalan Institute of Statistics [49]. The financial, social health, and macroeconomic data refer to the 2006–2017 period.

The data processing and analysis were performed using the IBM SPSS version 24.0 statistical package for Windows. First, a descriptive and graphic analysis of the variables was carried out. Second, a hypothesis test was performed to corroborate whether the main variables of our work were independent with respect to the variables that were used as factors. That is, the means of the distributions of the quantitative variable in the different groups established by the categorical variable were compared. 

To prove the normality assumption of the variables, we used the Shapiro–Wilk test, since the number of data points is under 50 for each of the communities (Table 2).

According to this test, for the case of region 1, all the variables except for asset turnover behaved normally, since their significance is over 0.05. In region 2, cash flow did not behave normally. By contrast, the variables of GDP per capita, asset turnover, and expenditures per inhabitant did behave normally. We thus decided to use parametric variables, since only 2 variables out of all the dependents were not normally distributed.

For the estimation of the regression model, considering the expenditure per capita as the dependent variable, those variables that presented a high and very high correlation were included, with an r > 0.6 (Table 1, Table 2, Table 3, 5 and 6). Previously, all the assumptions needed to perform a regression analysis were tested. The mean variance inflation factor (VIF) was very close to 1 (1.155) and there was no VIF greater than 10, which proved non-multicollinearity. Regarding error independence, the value of the Durbin–Watson test was 2.08, which corroborates independence as the value was between 1 and 3.

A determination coefficient was obtained from the executed SPSS orders, this being the proportion of the total variance of the variable explained by the regression, reflecting the model’s goodness of fit with the dependent variable that was intended to be explained. For this reason, those that gave an R² greater than 0.6 and a significant ANOVA *p*-value <0.05 were chosen as predictor variables, as we were able to affirm that the predictor variables obtained in the regression were good at explaining the dependent variable, expenditure per capita.

## 3. Results

According to the results obtained in the descriptive analysis, significant differences were found between both regions under study. The average expenditure per capita for the region where the management by agreement prevails was 1314 EUR/inhabitant, while for the region where the centers are publicly owned it was 1575 EUR/inhabitant (Table 3 and Table 4).

In terms of mental health expenditure per capita, we were able to verify that in those regions where the health system is managed by public ownership, more is invested than in the regions where it is managed by agreement. Furthermore, the standard deviation of the former was much higher, which indicates that in the latter there is a lower but more equal expenditure per patient.

The mean of the expenditure per capita variable was higher in the region that is managed by public ownership throughout the period analyzed. Furthermore, the difference between the means of both regions increased throughout the period.

The next table shows the correlation analysis.

According to the information presented in the correlation tables (Table 5 and Table 6), in the first region, there is significance in the correlation between expenditure per inhabitant with cash flow and GDP per capita, since its p-value is below 0.05 and the Pearson correlation coefficient is moderately high. In the second region, expenditure per inhabitant is significantly correlated with asset turnover, GDP per capita, and expenditure as % GDP. These correlations are also moderately high. This did not allow for selecting variables for the regression analysis.

The assumptions for the regression analysis were analyzed in the methodology (Table 7).

The ANOVA of the regression model with two variables indicated that it significantly improved the prediction of the dependent variable, with an F = 14.406 and a significance of less than 0.05. For the regression model tested with the two independent variables, the R2 explained 76.2% of the variance. The resulting equation of the multiple regression model was the following:

Expenditure per capita = 72.686 + 0.00001672. Cash Flow + 0.004. GDP per capita(1)

Regarding the region where direct management prevails, the execution of the test is shown in Table 8:

Next, we considered the fourth model with a coefficient of determination of 0.884, and with this, it was determined that the variables expenditure as a percentage of GDP and asset turnover, together, were good at explaining around 88% of the variability of the dependent variable.

The ANOVA of the regression model with two variables indicated that it significantly improved the prediction of the dependent variable, with an F = 34.293 and a significance of less than 0.05. For the regression model tested with the two independent variables, R2 explained 88.4% of the variance. The resulting equation of the multiple regression model for the region with direct management is the following:

Expenditure per capita = −258.862 + 251.866. Asset Turnover + 257.851. Exp as % GDP(2)

## 4. Discussion

The objective of this study is to observe the influence of different macroeconomic and financial variables on per capita spending within a mental health framework, whether in regions with healthcare under public title or in regions with concerted management. The results are interesting, since they show some significant differences between both models, and economic activity in GDP terms has a greater impact in regions with a public system. Business management in terms of results is more relevant in the health system managed in concert.

Both for regions that have opted for the agreement formula and for those that have opted for direct management, the hypothesis was fulfilled with 95% confidence, since significant differences were found in the mean between the variables. The average expenditure per capita for the entire period studied was 1314.72 EUR/inh. in the region where management by agreement prevails, and 1575.46 EUR/inh. in the region with direct management.

If we consider the temporal evolution of the region where the healthcare model is managed by agreement, it can be seen that from 2006 to 2010 the expenditure per capita increased. Then, from 2011 to 2013 a decrease occurred due to the financial crisis, with the year 2012 being the highest decrease in expenditure per capita, representing 6.67% compared to the previous year. In 2014, the expenditure per capita practically did not change compared to 2013, and then again suffered a considerable increase of 7.22% in 2015.

Then, if we consider the temporal evolution of the region where the health system is managed directly, it can be seen that from 2006 to 2009 there was an increase in the expenditure per capita of more than 7%. Then, from 2009 to 2013 there was a decrease, although in no year was it greater than 3.4%. After 2013, the expenditure per capita increased again each year, but in no case was this annual increase comparable to that of the 2006–2009 period.

Therefore, although in both regions the financial crisis affected the expenditure per capita, in the years prior to the crisis it was the region in which the health system is directly managed that presented the most significant increases; and it is also the community that, in the face of a crisis, decreases the expenditure per capita less. Although it is true that after the financial crisis (after 2013) there was an increase in the expenditure per capita in both regions, this was higher in the region where management by agreement prevails. However, throughout the period studied, the expenditure per capita was lower for this region.

Although mental health expenditure in the region where the agreement is used is lower than in the region with direct management, it is more egalitarian, that is, there are not so many differences in terms of the service provided, the standard deviation being 84.814. On the contrary, in the region with publicly owned management, where the expenditure is higher, there is more difference in expenditure per patient, with a standard deviation of 123.666.

We found that for the regions where the agreement prevails, two crucial variables for expenditure per capita on mental health are the GDP per capita and the cash flow of the mental health provider centers. On the other hand, for the region with direct management, the variables asset turnover of providers in mental health and mental health expenditure as a percentage of GDP per capita are crucial.

An interesting conclusion is that the economic activity of the regions affects expenditure on mental health [50,51]. This means that, as the regions grow in production, investment in mental health improves.

Therefore, from the equation of the multiple regression model, the cash flow is considered as a predictor variable for the regions with agreement, which is a financial variable that also considers the economic result of the mental health provider companies and the GDP.

The equation of the multiple regression model for the region that is managed directly considers the asset turnover and the expenditure as a percentage of GDP as predictor variables. The first is a financial variable that is affected by the investments made; in other words, the greater the investment in assets, while keeping the sales/income constant, the lower the asset turnover.

The strength of the present study lies in how the results obtained can help health policy managers from different countries or various regions within a country carry out healthcare policies within a mental health framework, which is the objective of the present study, always bearing the concepts of efficiency and equity in mind.

This work is not without limitations. The analysis was carried out until the year 2017 since it was the last year for which data was available. In the future, it will be key to study more regions and international cases and compare them with the current study.

Although it is true that there are previous studies that relate health expenditure to GDP, there are no comparative studies between regions where indirect management prevails and regions where direct management prevails that integrate macroeconomic and financial variables in the mental health framework.

One of the future lines of this work will be to consider the impact that COVID-19 has had on mental health expenditure per capita since in the current context, we are faced with an unprecedented situation.

Another good future line of research in light of the available data would be to show the significant annual differences. For this, we would need monthly data, while the currently available data are annual.

## 5. Conclusions

This study, for the first time in the scientific literature, concludes that it is expected that in periods of low economic activity, that is, when GDP is reduced, the “expenditure per capita” will also be reduced. This situation was experienced during the financial crisis, the results of which have been exposed in this study, and also during the current COVID-19 pandemic.

Thus, the reduction in GDP and in expenditure per capita will affect the service that can be offered to people who suffer from a mental illness and/or the salaries of health personnel, as well as the prices paid to mental health supplier companies.

In the case of direct management, it is easier to make a smaller reduction to the expenditure per capita even if public administrations go into debt since these companies are publicly owned.

Regarding indirect management, an important factor to take into account is that the supplier companies seek profitability; therefore, there is a decrease in the expenditure per capita. The results suggest that local administrations should consider other parameters when investing in mental health and should consider what the burden of disease represents in the countries.

In conclusion, we can show some significant differences between both models. As we can expect, in those regions with concerted operations, the economic results of the mental health provision companies are a highly important factor. The economic management of these centers is also a determining factor. These companies’ results can also be compromised by certain health policies, such as what happened during the 2009–2013 crisis.

These results are useful for presenting the need for investment in order to carry out health policies that can achieve equity and efficiency in the system, as well as definitively improving health services, particularly the mental health services which concern us here, given that higher per capita mental health spending will provide more services to the general population.

In this sense, the current crisis caused by COVID-19 has shown important deficiencies in health systems and a rather reduced investment in mental health.

## Figures and Tables

**Table 1:** Description of variables.

Variables	Description
Asset Turnover	Income/sales generated in relation to the investment in assets
Cash Flow	Net profit plus the amortizations
GDP Per Capita	GDP per capita (or GDP per inhabitant), is the result of dividing GDP by the number of inhabitants
Expenditure Per Inhabitant	Expenditures from the public sector, both in acquiring goods and services and in providing services
Expenditure as % GDP	The weight of public spending on GDP

**Table 2:** Normality test. Region 1 with management by agreement. Region 2 with direct management.

Normality Test							
Regions		Kolmogorov–Smirnov ^a^			Shapiro-Wilk		
		Statistics	gl	Sig.	Statistics	gl	Sig.
1	Asset Turnover	0.256	12	0.029	0.858	12	0.046
	Cash_flow	0.210	12	0.152	0.937	12	0.461
	GDP Per Capita	0.133	12	0.200 *	0.956	12	0.725
	Expenditure as % GDP	0.163	12	0.200 *	0.946	12	0.576
2	Asset Turnover	0.168	12	0.200 *	0.940	12	0.510
	Cash_flow	0.344	12	0.000	0.647	12	0.000
	GDP Per Capita	0.137	12	0.200 *	0.948	12	0.610
	Expenditure as % GDP	0.243	12	0.548	0.836	12	0.525

^a^. Lilliefors significance correction; *. This is a lower limit of true significance.

**Table 3:** Descriptive statistics model for regions with management by agreement.

	Mean/Std. Error	Median	Variance	Std. Deviation	Interquartile Range
Expenditure Per Inhabitant	1314.72/24.48	1340.53	7193.495	84.81	137.07
Asset Turnover	0.956/0.0284	0.935	0.010	0.098	0.185
Cash Flow	26,302,103.25/904,381.830	26,793,509.00	9,814,877,937,676.205	3,132,870.559	3,370,855
GDP Per Capita	201,597.58/2651.217	200,923.00	84,347,438.083	9184.086	12,947
Expenditure as % GDP	4.77/0.085	4.758	0.087	0.295298734	0.377169

**Table 4:** Descriptive statistics model for regions with direct management.

	Mean/Std. Error	Median	Variance	Std. Deviation	Interquartile Range
Expenditure Per Inhabitant	1575.46/35.69	1606.69	15,293.350	123.66	107.523718
Asset Turnover	1.928/0.041168737775	0.0411	0.020	0.142	0.267
Cash Flow	31,643,493.83/ 4,276,898.160	4,276,898.160	219,502,294,447,988.700	14,815,609.824	6,928,505
GDP Per Capita	29,713.08/335.881	29,509.50	1,353,795.720	1163.527	1867
Expenditure as % GDP	5.230/0.104	5.385	0.130	0.360	0.438067

**Table 5:** Correlation matrix of variables for regions with management by agreement.

Correlations							
Model for Regions where Management by Agreement Prevails			Asset Turnover	Cash Flow	GDP Per Capita	Expenditure Per Inhabitant	Expenditure as % GDP
Spearman’s Rho	Asset Turnover	Correlation coefficient	1.000	0.063	0.294	0.343	−0.070
		Sig. (bilateral)		0.846	0.354	0.276	0.829
		N	12	12	12	12	12
	Cash Flow	Correlation coefficient	0.063	1.000	0.350	0.643 *	0.413
		Sig. (bilateral)	0.846		0.265	0.024	0.183
		N	12	12	12	12	12
	GDP Per Capita	Correlation coefficient	0.294	0.350	1.000	0.629 *	−0.119
		Sig. (bilateral)	0.354	0.265		0.028	0.713
		N	12	12	12	12	12
	Expenditure Per Inhabitant	Correlation coefficient	0.343	0.643 *	0.629 *	1.000	0.552
		Sig. (bilateral)	0.276	0.024	0.028		0.063
		N	12	12	12	12	12
	Expenditure as % GDP	Correlation coefficient	−0.070	0.413	−0.119	0.552	1.000
		Sig. (bilateral)	0.829	0.183	0.713	0.063	
		N	12	12	12	12	12

* Correlation is significant at the 0.05 level (bilateral).

**Table 6:** Correlation matrix of variables for regions with direct management.

Correlations							
Model for Regions with Direct Management			Asset Turnover	Cash Flow	GDP Per Capita	Expenditure Per Inhabitant	Expenditure as % GDP
Spearman’s Rho	Asset Turnover	Correlation coefficient	1.000	0.014	0.546	0.776 **	0.329
		Sig. (bilateral)		0.966	0.066	0.003	0.297
		N	12	12	12	12	12
	Cash Flow	Correlation coefficient	0.014	1.000	−0.301	0.084	0.580 *
		Sig. (bilateral)	0.966		0.341	0.795	0.048
		N	12	12	12	12	12
	GDP Per Capita	Correlation coefficient	0.546	−0.301	1.000	0.539	−0.028
		Sig. (bilateral)	0.066	0.341		0.070	0.931
		N	12	12	12	12	12
	Expenditure Per Inhabitant	Correlation coefficient	0.776 **	0.084	0.539	1.000	0.671 *
		Sig. (bilateral)	0.003	0.795	0.070		0.017
		N	12	12	12	12	12
	Expenditure as % GDP	Correlation coefficient	0.329	0.580 *	−0.028	0.671 *	1.000
		Sig. (bilateral)	0.297	0.048	0.931	0.017	
		N	12	12	12	12	12

* Correlation is significant at the 0.05 level (bilateral). ** Correlation is significant at the 0.01 level (bilateral).

**Table 7:** Summary of the model for regions where management by agreement prevails. Dependent variable: expenditure per inhabitant.

Model Summary				
Model	R	R^2^	Adjusted R^2^	Standard Error of the Estimate
2	0.873 ^b^	0.762	0.709	45.745740877

^b^ Predictors: (Constant), GDP_PER_CAPITA, Cash_flow.

**Table 8:** Summary of the model for regions with direct management. Dependent variable: expenditure per inhabitant.

Model Summary				
Model	R	R^2^	Adjusted R^2^	Standard Error of the Estimate
4	0.940 ^d^	0.884	0.858	46.564318114

^d^ Predictors: (Constant), GTO % PIB, ASSET_turnover.

## Data Availability

Not applicable.
